# Cerebral Venous Thrombosis and Nitrous Oxide Intoxication: Report of Two Cases and Review of the Literature

**DOI:** 10.1002/brb3.70394

**Published:** 2025-02-28

**Authors:** Diana Doukhi, Virginie Siguret, Dominique Vodovar, Maxime Delrue, Peggy Reiner, Agnes Aghetti, Stéphanie Guey, Mikael Mazighi, Isabelle Crassard

**Affiliations:** ^1^ Neurology Department Hôpital Lariboisière, APHP Paris France; ^2^ FHU NeuroVasc Paris France; ^3^ Université Paris Cité Paris France; ^4^ INSERM, U1140 Paris France; ^5^ Hematology Laboratory Department Hôpital Lariboisière, APHP Paris France; ^6^ Médecine Intensive Réanimation, Hôpital Charles Nicolle Rouen France; ^7^ U.F.R. de médecine Université Rouen Normandie Rouen France; ^8^ INSERM, U1096 Université Rouen Normandie Rouen France; ^9^ INSERM, U1144 Paris France; ^10^ Centre de Référence des Maladies Vasculaires Rares du Cerveau et de l'Oeil (CERVCO) Hôpital Lariboisière, APHP Paris France; ^11^ Interventional Neuroradiology Department Hôpital Fondation A. de Rothschild Paris France

**Keywords:** cerebral venous thrombosis, drug, homocysteine, nitrous oxide, vitamin B12

## Abstract

**Background:**

Cerebral venous thrombosis (CVT) has various risk factors, including contraception, pregnancy, neoplasia, and thrombophilia. Nitrous oxide (N₂O), historically used as an anesthetic and more recently as a recreational drug, has been associated with neurological complications such as myeloneuropathy and thromboembolic events. Here, we report two cases of CVT associated with N₂O use and provide a review of the literature on this association.

**Methods:**

We describe two local cases of CVT associated with N_2_O use and 10 additional cases identified by literature review.

**Results:**

Among the 12 patients, seven had co‐existing CVT risk factors. Most patients reported chronic N_2_O use. Hyperhomocysteinemia was reported in nine patients. Management included anticoagulation, vitamin supplementation, cessation of N₂O use, and support for addiction or psychiatric care. The outcome was generally favorable, although one local case experienced CVT recurrence following a relapse in N₂O use.

**Conclusions:**

These cases highlight an emerging association between CVT and N₂O use. Prompt recognition of this link is critical to recommend cessation of N₂O use, alongside anticoagulation therapy and consideration of vitamin supplementation to prevent complications and recurrence.

## Graphical abstract

This manuscript presents two well‐characterized cases of CVT linked to N_2_O misuse, managed at a French reference center for rare cerebrovascular diseases. To contextualize our findings, we also provide a narrative review of the existing literature, identifying 10 additional cases of CVT associated with N_2_O use. Our review highlights common clinical features, mechanisms, and management strategies for this rare but significant association.

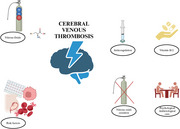

## 1 Introduction

Cerebral venous thrombosis (CVT) is a relevant cause of stroke in young adults, with an estimated incidence of 1.3 per 100,000 person‐years (Coutinho et al. 2012). Several risk factors have been identified, including oral contraceptive use, pregnancy and the postpartum period, neoplasia, anemia, thrombophilia (Silvis et al. 2017), and hyperhomocysteinemia (Cantu et al. 2004; Duman et al. 2017; Triquenot Bagan et al. 2021; Green et al. 2018).

Nitrous oxide (N_2_O), discovered by Joseph Priestly in 1772 and known as “laughing gas,” was initially used as an anesthetic. Its applications were expanded to commercial and industrial sectors including use in food production. Simultaneously, N_2_O became popular as a recreational drug, due to its psychoactive effects, low cost, wide availability, legal status, and perceived safety. Recent years have witnessed a sharp rise in its recreational use, with the Global Drug Survey 2021 reporting a lifetime prevalence of 22.5%, and making it the 14th most commonly used substance globally (Winstock et al. 2021). This increasing N_2_O use has raised concerns due to its association with neurologic complications, particularly myeloneuropathy (Swart et al. 2021; Caré et al. 2022). More recently, serious thrombotic events, including venous and arterial localizations, have been described in association with N_2_O use (Guerlais et al. 2023; Caris et al. 2023). In a study analyzing medical records of N_2_O users at a large academic medical center, the prevalence of thrombotic events was estimated to be 5% (Caris et al. 2023).

In this article, we present two cases of recreational N_2_O users who developed CVT and provide a review of the literature on this specific emerging association.

## 2 Methods

### 2.1 Case Reports

The cases were summarized based on the available clinical and paraclinical data of two patients admitted in September 2021 and November 2023, respectively. Both patients were subsequently followed at our department (French reference center for rare cerebrovascular diseases, Lariboisiere, Paris, France) until August 2024. Nonopposition to the publication of clinical data for research purposes was obtained from both patients, in accordance with our local standard protocol.

### 2.2 Literature Search

We performed a comprehensive search of the PubMed database, up to August 2024, using the following search terms: (“nitrous oxide,” AND “cerebral venous thrombosis,” OR “venous thrombosis,” OR “neurological,” OR “neurologic.”). The search was limited to articles in the English language published in the past 5 years. Titles and abstracts were screened for eligibility. Full‐text articles were reviewed if they met the inclusion criteria, which encompassed observational studies, case reports, and case series. Additional sources cited within the references of these articles were also examined. We excluded review articles and studies not explicitly addressing cerebrovascular complications associated with N_2_O use.

## 3 Results

### 3.1 Case Presentations

#### 3.1.1 Case 1

A 26‐year‐old male was admitted to the emergency department in September 2021 for a severe, holocranial headache that progressively localized to the left side. The headache, which began earlier that morning, was accompanied by nausea, vomiting, and phono‐photophobia. The patient had no remarkable medical history and was not taking regular medication. He had received his second dose of the COVID‐19 vaccine SPIKEVAX (Moderna mRNA‐1273) 12 days before. He reported a smoking habit and frequent alcohol consumption.

**FIGURE 1:**
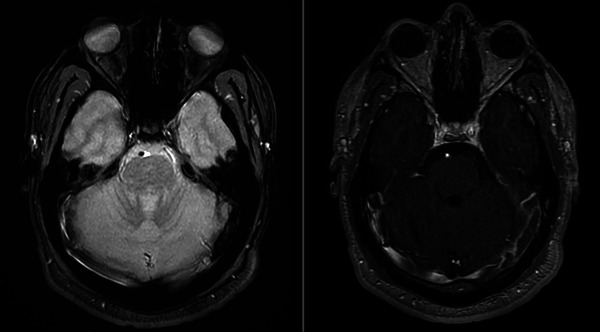
Brain MRI showing left lateral sinus thrombosis. (A) Axial T2* scan showing hypointensity in the left lateral sinus indicating the thrombus. (B) T1 with gadolinium showing an endoluminal defect in the left lateral sinus indicating thrombosis.

Neurological examination was unremarkable. A CT scan, followed by an MRI, revealed venous thrombosis of the left transverse sinus, sigmoid sinus, and jugular vein without associated parenchymal complications (Figure 1). Laboratory findings showed elevated homocysteine plasma levels at 97.1 µmol/L (reference values < 15), with normal vitamin B9 (4.3 µg/L, reference range: 3.9–20) and B12 levels (284 pmol/L, reference range: 140–664). D‐dimer level was elevated at 1110 ng/mL (reference values < 500). Complete blood count and C‐reactive protein were normal. Thrombophilia testing including the search for protein C, protein S, antithrombin deficiency, prothrombin gene (*F2)* G20210A and *F5* Leiden G1691A mutation, and antiphospholipid antibodies was also negative. Testing for the *MTHFR* C677T mutation was negative. Cerebrospinal fluid analysis was normal. There was no clinico‐biological feature suggesting any previous infection or current neoplasia.

The patient was initially treated with subcutaneous enoxaparin (100 IU/kg b.i.d.) for 5 days, then with oral dabigatran etexilate 150‐mg b.i.d. The patient's condition improved, and he was discharged symptom‐free. At 1‐month follow‐up, he admitted a regular recreational use of N_2_O at high doses during social events, a habit he had stopped after the CVT episode. He also reported prior episodes of transient paresthesia in the upper limbs during N_2_O use. Oral B9 and B12 supplementation was initiated. His homocysteine level was measured at 49.2 µmol/L in March 2022. The patient was subsequently lost to follow‐up.

In August 2022, the patient was re‐admitted with a recurrence of a left‐sided headache and nausea persisting for 5 days. He had stopped the dabigatran therapy on his own in February 2022 and continued N_2_O use, two to three times per week. MRI showed a new thrombosis in the left transverse sinus. Homocysteine levels remained elevated at 76.7 µmol/L, with low B9 level (3.7 µg/L) and normal B12 (322 pmol/L). Curative anticoagulant therapy with dabigatran etexilate 150‐mg b.i.d. was restarted, and the patient was again symptom‐free upon discharge.

#### 3.1.2 Case 2

A 21‐year‐old male presented to the emergency department in November 2023 with an intense, left‐sided headache accompanied by nausea, vomiting, phono‐photophobia, sensation of left ear fullness, and pharyngeal pain. Symptoms had been evolving for 1 week. The patient had a history of migraine but no other significant medical conditions and was not taking any regular medication. He reported a history of smoking, occasional alcohol consumption, and prior cannabis use.

**FIGURE 2:**
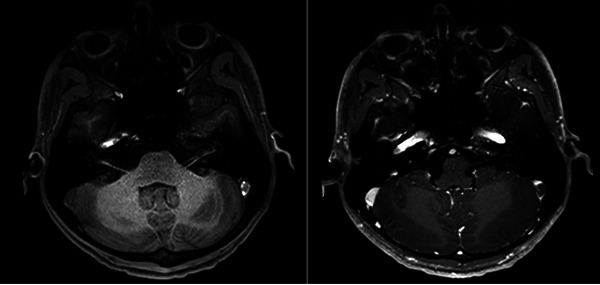
Brain MRI showing left sigmoid sinus thrombosis. (A) Axial T1 scan showing spontaneous hyperintensity in the left sigmoid sinus indicating the thrombus. (B) Axial T1 scan after gadolinium injection showing an endoluminal defect in the left sigmoid sinus indicating thrombosis.

Neurological examination was unremarkable. A CT scan, followed by an MRI, revealed CVT of the left transverse sinus, sigmoid sinus, and internal jugular vein without parenchymal lesions (Figure 2). Laboratory testing revealed a slightly elevated homocysteine level (34.3 µmol/L), with normal B9 (4.4 µg/L) and B12 (158 pmol/L) levels. Lupus anticoagulant was initially present (ratio: 1, 23, reference values < 1, 20) but was negative on repeat testing after 12 weeks. Screening for anti‐cardiolipin and anti‐beta2‐glycoprotein‐I antibodies was negative, but anti‐phosphatidylethanolamine antibodies (IgM 45 U/mL, reference values < 20) were persistently positive. Complete blood count, protein C, protein S, and antithrombin activities were within normal limits, and testing *F2* G20210A and *F5* Leiden G1691A was negative. The patient was heterozygous for the *MTHFR* C677T variant.

Cerebrospinal fluid analysis was unremarkable. C‐reactive protein was initially elevated at 19 mg/L (reference values < 5) with concomitant pharyngeal and ear complaints; however, ENT evaluation, repeated CRP tests, and a local CT scan were normal. There was no clinical suspicion of other previous infections or current neoplasia.

The patient was initially treated with subcutaneous enoxaparin (100 IU/kg b.i.d.), then switched to warfarin. His condition improved, with complete resolution of the headache, and he was discharged. During follow‐up, elevated homocysteine levels persisted, and the patient admitted chronic use of N_2_O, though the quantity of use was not specified. Oral B9 and B12 supplementation was then initiated. He showed no recurrence until the last follow‐up in August 2024.

### 3.2 Literature Review

The literature review identified 10 other cases of CVT associated with N_2_O use (Table 1) (Caris et al. 2023; Pratt et al. 2020; Liu et al. 2020; de Valck et al. 2021; Farhat et al. 2022; Lin et al. 2022; Peng et al. 2023; Banjongjit et al. 2023; Er et al. 2023; Patyjewicz et al. 2024). Patients were mainly young adults, with a median age of 21 years (ranging from 16 to early 30 years of age), and a relatively balanced gender distribution. Most patients reported chronic N₂O use, spanning from months to years, with no cases associated with first‐time single exposure. Several patients reported daily or frequent use of large amount of N₂O.

**TABLE 1:** Reported cases of cerebral venous thrombosis associated with nitrous oxide use.

Study	Pratt et al. (2020)	Liu et al. (2020)	De Valck et al. (2021)	Farhat et al. (2022)	Lin et al. (2022)	Peng et al. (2023)	Caris et al. (2023)	Banjongjit et al. (2023)	Er et al. (2023)	Patyjewicz et al. (2024)	Case 1	Case 2
Age/Gender	21 F	25 F	24 M	16 F	25 M	20 M	18 F	19 F	21 F	30	26 M	21 M
Relevant history	Elective abortions	None	None	None	Recurrent PE, B12 deficiency	NR	None	None	None	None	None	Migraine
Nitrous oxide habit	600 vials/day for at least several weeks	For 20 months	Shared 925 balloons/week for multiple weeks	Up to 18 whippets /day for at least 3 weeks	NR	For 3 months	1 shared cylinder (2000 g)/2 weeks for 1 year	320 g/day for 6 months and 2880 g/day for 1 month	2–4 canisters/day for at least 1 month	5–6 cylinders/3 weeks for 3 years	High dose 2–3 times/week	Chronic use without quantification
Other risk factors	Pregnancy 11‐week gestation, severe pancytopenia	Oral contraceptive	Post dural puncture headache, heterozygote for *F2* G20210A	COVID‐19 6 weeks prior	Recurrent PE, Astra‐Zeneca vaccine 1 month prior without VITT	NR	Oral contraceptive	None	None	None	None	Persistent antiphosphatidylethanolamine antibodies
Thrombus location	Left LS SS and JV	Left CV	SSS	SSS, StS, bilateral LS, right SS and JV, SV and DCV	StS, bilateral LS	SSS, left LS and SS	Left JV, SS, bilateral LS	SSS, bilateral, DCV, vein of Galien, StS, bilateral LS, left SS, left, CV	NR	Right LS and SS	Left LS SS and JV	Left LS SS and JV
Parenchymal lesion	None	Hemorrhagic infarction/subarachnoid hemorrhage in the left parietal lobe	Left fronto parietal ischemia	Deep brain ischemic lesions	None	Left temporal lobe infarction with nodal hemorrhage inside	Hemorrhagic infarction in the left parietal and occipital lobes	Several intraparenchymal hematomas: Left frontal, right parietal, right parietotemporal	None	None	None	None
Homocysteinemia (µmol/L)	↑, 65	N, 14	N (after B12)	↑, 134	↑, 59	↑, 26	↑, 69	↑, 101	↑, 58	NR	↑, 97	↑, 34
B12 (pmol/L)	N	N	↓, 81	↓, 69	↓, <109	N, 179	N, 175	↓, <74	↓, 63	N, 245	N, 264	N, 158
Treatment: Anticoagulation B12 Other treatments	UFH Yes B9	LMWH then oral anticoagulant Yes B6, B9, mannitol	LMWH then dabigatran Yesd Valproic acid, acetazolamide	Heparin Yes B9, acetazolamide	LMWH then edoxaban Yes B6, B9, levetiracetam, mannitol	Warfarin Yes B9, diazepam valproic acid, mannitol	LMWH then dabigatran Yes	UFH then LMWH, then warfarin 3 months Yes Diazepam, levetiracetam, lamotrigine, dexamethasone, mannitol	LMWH then rivaroxaban NR	LMWH NR	LMWH then dabigatran Yes B9	LMWH then warfarin Yes B9
Outcome	Significant improvement in gait and weakness	Significant improvement in weakness to grade 4/5, and verbal fluency	Chronic intracranial hypertension, short‐term memory issues, and fatigue at 9 months	No sequelae	Significant improvement in headache and quadranopsia	No sequelae	NR	Significant improvement in weakness	NR	No sequelae	No sequelae	No sequelae
Recurrence	NRs	None at 6 weeks	None at 9 months	None at 6 months	None at 7 days	None at 9 months	NR	NR	NR	NR	Yes at 11 months	None at 7 months
Associated myeloneuropathy	None	Yes	Yes	None	None	None	None	Yes	None	None	Possible	None

Abbreviations: CV, cortical vein; DCV, deep cerebral veins; F, female; JV, jugular vein; LMWH, low molecular weight heparin; LS, lateral sinus; M, male; NR, not reported; PE, pulmonary embolism; SS, sigmoid sinus; SSS, superior sagittal sinus; StS, straight sinus; UFH, unfractionated heparin; VITT, vaccine‐induced immune thrombocytopenia; ↑, increased; ↓, decreased.

Clinically, patients were most presented with headaches. Seizures were noted in four cases (Caris et al. 2023; de Valck et al. 2021; Peng et al. 2023; Banjongjit et al. 2023). A few cases reported the association with previous or concomitant N₂O‐induced myeloneuropathy (Liu et al. 2020; de Valck et al. 2021; Banjongjit et al. 2023, Case 1). CVT locations varied, most commonly affecting the superior sagittal and lateral sinuses. Parenchymal complications, such as hemorrhagic infarctions or ischemic lesions, were present in half of the cases (Caris et al. 2023; Liu et al. 2020; de Valck et al. 2021; Farhat et al. 2022; Peng et al. 2023; Banjongjit et al. 2023). Hyperhomocysteinemia was reported in nine out of twelve cases with a median homocysteine level of 65 µmol/L (range: 26–134). Among the remaining three cases, homocysteine levels were normal at admission in one (Liu et al. 2020), normal after B12 supplementation in another (de Valck et al. 2021), and not reported in the last case (Patyjewicz et al. 2024). Of those with hyperhomocysteinemia, five had normal vitamin B12 levels despite elevated homocysteine. Additional CVT risk factors were identified in seven of twelve cases, including pregnancy (Pratt et al. 2020), oral contraceptive use (Caris et al. 2023; Liu et al. 2020), COVID‐19 vaccination (Lin et al. 2022), heterozygous *F2* G20210A mutation (de Valck et al. 2021), and the presence of antiphospholipid antibodies (Case 2). All patients received anticoagulation therapy, along with B12 supplementation. Other treatments included antiepileptic drugs (Er et al. 2023; Chanarin 1980; den Heijer et al. 1996; Ray et al. 2007), acetazolamide (de Valck et al. 2021; Farhat et al. 2022), mannitol (Liu et al. 2020; Peng et al. 2023; Banjongjit et al. 2023), and B6 and B9 supplementation (Pratt et al. 2020; Liu et al. 2020; Farhat et al. 2022; Lin et al. 2022; Peng et al. 2023, our cases). N_2_O withdrawal was recommended.

The outcome was generally favorable, with most patients experiencing significant recovery. Recurrence of CVT was documented in only one case (Case 1), which was potentially associated with the resumption of N₂O use.

## 4 Discussion

Our two cases, combined with the literature review, illustrate a rare but emerging association between recreational N_2_O use and CVT. Notably, we report a case of CVT recurrence in the setting of persistent N_2_O consumption. As N_2_O has become increasingly popular as a recreational drug, particularly among young adults, growing evidence highlights the potential neurological complications related to its use. Early recognition and management of these complications are essential to prevent irreversible neurological damage and recurrent thrombotic events.

Historically, the neurological complications of N_2_O use have been predominantly linked to its impact on vitamin B12 metabolism. The most well‐documented consequence is myeloneuropathy, presenting with sensory disturbances such as paresthesia, ataxia, and gait instability (Swart et al. 2021). It is attributed to the inactivation of vitamin B12 (cobalamin), caused by N₂O irreversibly oxidizing the cobalt ion in B12, rendering it biologically inactive (Chanarin 1980). As biologically active B12 is an essential cofactor of methionine synthase, an enzyme responsible for converting homocysteine to methionine, N₂O‐induced B12 inactivation is responsible for decreased production of myelin, contributing to the demyelination seen in these patients. Concurrently, the accumulation of homocysteine results in hyperhomocysteinemia.

In the setting of N_2_O‐associated CVT, this resulting hyperhomocysteinemia may contribute as a risk factor, although its role remains controversial. Hyperhomocysteinemia has been associated with the onset of thrombotic events, including CVT (Cantu et al. 2004; Duman et al. 2017; Triquenot Bagan et al. 2021; Green et al. 2018). The primary hypothesis points to its toxic effects on the vascular endothelium and disruption of the coagulation cascade (Chanarin 1980). However, randomized trials have not demonstrated a clear benefit of homocysteine‐lowering therapy on the risk of initial or recurrent venous thrombosis (Ray et al. 2007; den Heijer et al. 2007).

Importantly, not all N₂O users with CVT have hyperhomocysteinemia, suggesting that additional risk factors may play a role. Some patients exhibit other established risk factors for CVT, such as oral contraceptive use, pregnancy, or underlying thrombophilia, which may act as confounding factors. Additionally, not all individuals who develop myeloneuropathy with hyperhomocysteinemia following N₂O use go on to develop CVT. This highlights the possibility that additional patient‐specific risk factors or co‐factors may play a role in the pathogenesis of CVT.

Identifying N₂O use can be challenging, as patients may be reluctant to disclose their consumption, especially if not explicitly questioned, which may further complicate the diagnostic process. In both of our cases, N_2_O misuse was only revealed weeks after the initial presentation. Clinicians should actively inquire about N_2_O use in CVT‐suspected cases, particularly in young patients and/or those with a history of substance use, such as alcohol and cannabis. Asking direct but nonjudgemental questions can facilitate disclosure, for example: “*Have you ever used ‘laughing gas’, whippets, or balloons recreationally?” “Do you use any substance for relaxation or recreation?”*

In the absence of specific biomarkers, hyperhomocysteinemia may serve as a clue in clinical practice, even if B12 levels appear normal. Other potential indicators, such as methylmalonic acid, could also be considered (Pratt et al. 2020).

Finally, while curative anticoagulation remains the standard treatment for CVT, we propose that vitamin B12 supplementation should be considered in all patients with suspected N_2_O‐related CVT, despite the lack of specific recommendation or proven benefit. Further studies are needed to determine whether B12 supplementation influences clinical outcomes or CVT recurrence risk. The question of whether vitamin B12 supplementation should be routinely administered to patients misusing N_2_O remains open. Moreover, as most patients with complications related to N_2_O use are chronic users and likely addicted, they require specific care addressing this addiction. This should include a referral for addictological and/or psychiatric support.

In conclusion, with the increasing prevalence of N_2_O use, clinicians should consider it as a potential cause of CVT, especially when hyperhomocysteinemia is present, and independently of the presence or absence of associated thrombotic risk factors. Early identification, alongside specific treatments such as cessation of N_2_O use and consideration for B12 supplementation, is important to help prevent further complications and reduce the risk of recurrence. Further research is needed to establish evidence‐based recommendations for screening and management.

## Author Contributions

**Diana Doukhi**: conceptualization, methodology, formal analysis, investigation, writing – original draft. **Virginie Siguret**: writing – review and editing. **Dominique Vodovar**: writing – review and editing. **Maxime Delrue**: writing – review and editing. **Peggy Reiner**: resources. **Agnes Aghetti**: resources. **Stéphanie Guey**: resources. **Mikael Mazighi**: writing – review and editing, supervision. **Isabelle Crassard**: conceptualization, resources, supervision, writing – review and editing.

### Peer Review

The peer review history for this article is available at https://publons.com/publon/10.1002/brb3.70394.

## Data Availability

Data sharing is not applicable to this article as no datasets were generated or analyzed during the current study.
